# 缓冲盐类型和离子对试剂非对离子对强离解酸性化合物离子对反相液相色谱保留行为的影响

**DOI:** 10.3724/SP.J.1123.2021.06044

**Published:** 2021-09-08

**Authors:** Xiaolan LIU, Wei GAO, Chao LIANG, Junqin QIAO, Kang WANG, Hongzhen LIAN

**Affiliations:** 1.生命分析化学国家重点实验室, 南京大学化学化工学院, 南京大学现代分析中心, 江苏 南京 210023; 1. State Key Laboratory of Analytical Chemistry for Life Science, School of Chemistry & Chemical Engineering and Center of Materials Analysis, Nanjing University, Nanjing 210023, China; 2.泰州医药高新技术产业园区公共平台服务中心, 江苏 泰州 225300; 2. Taizhou Medical High-Tech Industrial Zone Public Platform Service Center, Taizhou 225300, China; 3.济川药业集团有限公司, 江苏 泰兴 225441; 3. Jumpcan Pharmaceutical Group Co., Ltd., Taixing 225441, China

**Keywords:** 离子对反相液相色谱, 缓冲盐类型, 离子对试剂非对离子, 保留行为, 定量结构-保留行为关系, ion-pair reversed-phase liquid chromatography (IP-RPLC), buffer salt type, non-counter ion of ion-pair reagent, retention behavior, quantitative structure-retention relationship (QSRR)

## Abstract

在离子对反相液相色谱(IP-RPLC)分析中,溶质保留受对离子(counter ion)的影响比较受人关注,但鲜有研究流动相中缓冲盐类型和离子对试剂中非对离子(non-counter ion)对溶质保留行为的影响。鉴于此,该文以14种磺酸化合物为研究对象,甲醇为有机调节剂,分别考察了3种缓冲盐体系(磷酸二氢铵、氯化铵和乙酸铵)和5种离子对试剂体系(四丁基溴化铵、四丁基磷酸二氢铵、四丁基硫酸氢铵、四丁基硝酸铵和四丁基乙酸铵)下强离解酸性化合物的IP-RPLC保留行为,通过比较不同流动相条件下得到的溶质log *k*_w_(100%水相作流动相时的保留因子)、*S*(线型溶剂强度模型线性回归得到的常数),以及CHI(色谱疏水指数,log *k*_w_/*S*),寻找保留行为规律。研究表明,流动相中的缓冲盐类型和离子对试剂非对离子均会影响化合物的log *k*_w_和*S*值,所有化合物在氯化铵缓冲盐体系下具有最大的log *k*_w_值。相对于无机阴离子,离子对试剂中弱离解性有机阴离子(乙酸根)的存在有利于增加磺酸化合物的*S*值。通过对比不同条件下的保留行为,推测磺酸化合物的IP-RPLC保留机理中同时存在着离子对模型和动态离子交换模型。与log *k*_w_和S值不同,化合物的CHI值受缓冲盐类型以及离子对试剂非对离子的影响较弱。此外,研究发现化合物的表观正辛醇/水分配系数(log *D*)与log *k*_w_、*S*、CHI之间均具有良好的线性相关性。不同缓冲溶液和不同离子对试剂非对离子条件下获得的log *k*_w_和*S*值存在着一定的差异,而CHI值相对稳定,因此,CHI更适用于IP-RPLC中定量结构-保留行为关系模型的建立。

## Abstract (en)

Ion-pair reversed-phase liquid chromatography (IP-RPLC) enhances separation by adding ion-pair reagents to the mobile phase, thereby improving the retention of oppositely charged solutes. IP-RPLC is primarily used for the separation and analysis of strongly ionized compounds. In IP-RPLC, researchers often focus more on the influence of the counter-ion type and concentration, buffer salt concentration and pH, and column temperature, on the retention behavior of solutes. However, the effects of the buffer salt type and non-counter ions in ion-pair reagents on the retention behavior of solutes have rarely been investigated. Accordingly, in this work, the effects of buffer salt types and non-counter ions on the retention behavior of strongly ionized compounds were investigated by IP-RPLC using 14 sulfonic acid compounds as model compounds. Experiments were performed using a silica-based C18 column with methanol as the organic modifier. In the first type of experiment, tetrabutylammonium bromide was kept unchanged as the ion-pair reagent in the mobile phase, and ammonium dihydrogen phosphate, ammonium chloride, and ammonium acetate were used as buffer salts, respectively. The retention factor (*k*) was obtained at different methanol ratios, and linear solvent strength (LSS) models were established to determine the log *k*_w_ (logarithm of retention factors of solutes when 100% aqueous phases were used as the mobile phase) and *S* (intercept of the LSS model) values of each solute. All solutes exhibited the highest log *k*_w_ with the ammonium chloride buffer system; most compounds also exhibited the highest *S* values with this system, except for 1,5-naphthalenedisulfonic acid, 4-methylbenzenesulfonic acid, 5-amino-2-nanphthalenesulfonic acid, and 4-hydroxybenzenesulfonic acid. However, the chromatographic hydrophobic indices (CHIs, log *k*_w_/*S*) of the solutes with different buffer salts were approximately equal. In the second type of experiment, ammonium dihydrogen phosphate was kept unchanged as the buffer salt in the mobile phase, and tetrabutylammonium bromide, tetrabutylammonium dihydrogen phosphate, tetrabutylammonium hydrogen sulfate, tetrabutylammonium nitrate, and tetrabutylammonium acetate were used as ion-pair reagents, respectively. Almost all solutes exhibited the highest *S* with the tetrabutylammonium acetate system, indicating that weakly ionized anions (such as acetate ions) in ion-pair reagents will improve the *S* values of sulfonic acid compounds. Interestingly, the CHIs of the solutes were almost the same for solutes with different non-counter ions. These results suggest that both, the buffer salt types and non-counter ions, influence the log *k*_w_ and *S* values of sulfonic acid compounds. Comparison of the retention behavior of solutes with different mobile phases suggested ion-pair mechanisms as well as dynamic ion-exchange mechanisms plays role in the IP-RPLC retention of sulfonic acid compounds. In addition, with all the experimental mobile phases, the apparent *n*-octanol/water partition coefficient (log *D*) presented a good linear correlation with log *k*_w_, *S*, and CHI, respectively, by the introduction of structure-related descriptors such as charge (*n*_e_), Abraham solvation parameters (*A* and *B*), and the polar surface area (PSA). Considering the differences in the log *k*_w_ and *S* values obtained with different buffer salts and non-counter ions, the CHIs were relatively stable; therefore, the CHI is more suitable for establishing a quantitative structure-retention relationship (QSRR) model in IP-RPLC, compared to log *k*_w_ and *S*.

反相液相色谱(RPLC)因具有良好的选择性,已成为当今应用最为广泛的色谱分离模式。然而,在分析一些离解性强的化合物时,单一的RPLC体系并不能得到满意的分离效果^[1]^。离子对反相液相色谱法(IP-RPLC)通过在流动相中添加离子对试剂,增强带相反电荷溶质的保留从而改善分离,主要用于强离解化合物的分离分析^[2,3,4,5,6,7]^。在IP-RPLC的应用研究中,科研工作者对影响化合物保留行为的因素一直颇为关注,开展了较为广泛的研究。Fletouris等^[8]^采用烷基季铵盐作为离子对试剂,对多种青霉素进行IP-RPLC分析,研究了配对离子链长度、流动相pH和浓度,以及柱温对青霉素保留行为的影响。Lu等^[9]^利用IP-RPLC梯度洗脱研究了氯雷他定及其8种相关化合物的保留行为与流动相pH以及离子对试剂浓度之间的关系。刘文霞等^[1]^将十二烷基硫酸钠(SDS)作为流动相添加剂,考察了不同色谱条件对表阿霉素及其6种相关物质保留行为的影响,为表阿霉素及其相关物质的分离测定提供了新思路。Burmaoglu等^[5]^采用蒸发光散射检测器,分别考察了离子对试剂浓度、有机调节剂类型、流动相pH、色谱柱类型、柱温等对唑来膦酸及其相关杂质保留行为的影响,建立了最佳分离分析策略。很显然,在IP-RPLC的应用研究中,人们主要关注离子对试剂的浓度及对离子(counter ion)类型、缓冲溶液的浓度及pH、柱温等对保留的影响,而离子对试剂中的非对离子(non-counter ion)以及缓冲盐类型对溶质保留行为的影响研究相对较少。

定量结构-保留行为关系(QSRR)模型可以沟通溶质保留与疏水性参数正辛醇-水分配系数(log *P*)之间的关系,可用于溶质保留行为的预测或者log *P*的测定^[10]^。对于离解性化合物,通常用表观正辛醇-水分配系数log *D*代替log *P*。研究^[11]^发现,在RPLC中以100%水相作流动相时的保留因子对数值(log *k*_w_)与log *P*或log *D*之间具有很好的相关性。Log *k*_w_可由线性溶剂强度(linear solvent strength, LSS)模型外推获得^[12]^:


(1) log *k*=log *k*_w_-*S*φ

*k*为溶质的保留因子,*φ*是流动相中有机调节剂的体积分数,*S*是线性回归得到的常数。

当溶质在流动相和固定相上达到相等分配(*k*=1, log *k*=0)时,对应的有机调节剂比例称为色谱疏水指数(chromatographic hydrophobicity index, CHI)。有文献^[13,14]^指出,在引入溶质氢键描述符的情况下,CHI与log *P*以及log *D*之间也存在着较好的线性关系。CHI可通过log *k*_w_和*S*计算^[15]^:


(2) CHI=log *k*_w_/*S*

本文以14种磺酸化合物为研究对象,在硅胶基质C18柱上,以甲醇为有机调节剂,采用IP-RPLC进行保留行为研究。首先,固定流动相中离子对试剂为四丁基溴化铵不变,考察缓冲盐类型(磷酸二氢铵、氯化铵和乙酸铵)对磺酸化合物保留行为的影响;然后,固定流动相中缓冲盐为磷酸二氢铵不变,考察四丁基季铵盐离子对试剂(四丁基溴化铵、四丁基磷酸二氢铵、四丁基硫酸氢铵、四丁基硝酸铵和四丁基乙酸铵)中阴离子对磺酸化合物保留行为的影响,并探索了IP-RPLC的保留机制。最后,对log *k*_w_、*S*、CHI与log *D*的相关性进行了比较。

## 1 实验部分

### 1.1 仪器、试剂与材料

实验所用高效液相色谱仪为Waters Alliance 2695(Waters,美国),仪器配有真空脱气机、数码四元泵和120位自动进样器。数据的采集和处理均在Waters Empower色谱管理系统中进行。用996紫外-可见二极管阵列(PDA)检测器在每个化合物的最佳吸收波长处检测其吸收峰。流动相pH值使用SevenMulti型pH/电导率/离子综合测试仪(Metter-Toledo,瑞士)测量。

实验中所用甲醇(HPLC级)购自美国Honeywell公司,所用水均为饮用纯净水(杭州娃哈哈集团);分析纯磷酸二氢铵、磷酸二氢钾、磷酸二氢钠、乙酸铵、氯化铵、氨水(25%~28%)、磷酸(85%)、冰乙酸(98%)和盐酸(36%~38%)均购自南京化学试剂股份有限公司;四丁基溴化铵(99%)购自百灵威公司(上海),四丁基硫酸铵(99%)购自安耐吉化学(上海),四丁基乙酸铵(98%)和四丁基磷酸氢铵(99%)购自毕得医药科技有限公司(上海),四丁基硝酸铵(99%)购自艾览化工科技有限公司(上海)。

本实验中对14种磺酸类化合物进行研究,具体信息见表1。其中,log *D*_7.0_值(pH=7.0条件下的log *D*值)是由本课题组前期工作中利用IP-RPLC测试得到的实验值^[16]^, p*K*_a_值由ACD/Labs软件计算得到,溶质静电荷*n*_e_、氢键酸性参数*A*、氢键碱性参数*B*、极性表面积PSA由https://ilab.acdlabs.com/iLab2/获取。磺酸化合物分别购自Accu Standard(美国)、TCI(日本)、国药集团化学试剂有限公司(上海)、Acros Organics(美国)、Matrix Scientific(美国)和Sigma-Aldrich(美国)。所有物质的纯度均大于98%,用甲醇配制成储备液(1.0 g/L),置于4 ℃冰箱中备用。

**表1:** 化合物的log *D*_7.0_、p*K*_a_、*n*_e_、*A*、*B*和PSA值

No.	Compound	log D_7.0_	pK_a_	n_e_	A	B	PSA/nm^2^
SA1	benzenesulfonic acid	-2.16	-0.60±0.50	-1.00	0.31	0.88	0.628
SA2	1,5-naphthalenedisulfonic acid	-4.69	-0.60±0.40	-2.00	0.63	1.71	1.260
SA3	4-chlorobenzenesulfonic acid	-1.08	-0.83±0.50	-1.00	0.31	0.87	0.628
SA4	4-methylbenzenesulfonic acid	-1.34	-0.43±0.50	-1.00	0.31	0.88	0.628
SA5	5-amino-2-nanphthalenesulfonic acid	-1.81	-0.23±0.40	-1.00	0.54	1.26	0.888
SA6	2-amino-1,4-benzenedisulfonic acid	-5.55	-1.15±0.50	-2.00	0.85	1.90	1.520
SA7	1-naphthalenesulfonic acid	-0.74	0.17±0.10	-1.00	0.31	0.94	0.628
SA8	2-naphthalenesulfonic acid	-0.75	0.27±0.10	-1.00	0.31	0.94	0.628
SA9	2,4-dimethylbenzenesulfonic acid	-1.09	-0.36±0.50	-1.00	0.31	0.89	0.628
SA10	4-sulfobenzoic acid	-4.70	-1.01±0.50	-2.00	0.88	1.21	1.000
SA11	3,5-dichloro-2-hydroxybenzenesulfonic acid	-0.33	-1.29±0.45	-1.14	0.81	0.90	0.830
SA12	3,5-dicarbomethoxybenzenesulfonic acid	-1.53	-1.34±0.30	-1.00	0.31	1.55	1.550
SA13	4-hydroxybenzenesulfonic acid	-2.70	-0.23±0.50	-1.01	0.81	1.15	0.830
SA14	3-sulfobenzoic acid	-4.36	-0.99±0.15	-2.00	0.88	1.21	1.000

log *D*_7.0_ (log *D* under pH 7.0) was obtained from our previous work^[16]^; p*K*_a_ (acidity coefficient) was calculated using ACD/Labs Software (V11.02, 1994-2021 ACD/Labs); *n*_e_, *A* and *B* values were obtained from https://ilab.acdlabs.com/iLab2/.

### 1.2 色谱条件

色谱柱:Welch Ultimate^®^ XB-C18(150 mm×4.6 mm, 5 μm,月旭科技(上海)股份有限公司);柱温:30 ℃;流速:1.0 mL/min;进样量:5 μL。各化合物的进样质量浓度均为50 mg/L,所有样品的保留时间(*t*_R_)均为至少3次独立进样的平均值。

流动相①: (甲醇+10 mmol/L四丁基溴化铵)-(20 mmol/L缓冲盐+10 mmol/L四丁基溴化铵,pH 7.0),分别使用磷酸二氢铵、氯化铵和乙酸铵作为缓冲盐。

流动相②: (甲醇+10 mmol/L四丁基季铵盐)-(20 mmol/L磷酸二氢铵+10 mmol/L四丁基季铵盐,pH 7.0),分别使用含不同阴离子的5种四丁基季铵盐(四丁基溴化铵、四丁基磷酸二氢铵、四丁基硫酸氢铵、四丁基硝酸铵和四丁基乙酸铵)作为离子对试剂。

### 1.3 实验方法

采用尿嘧啶测定死时间*t*_0_,所有化合物均使用等度洗脱,根据化合物疏水性差异,每个化合物至少在4个不同的甲醇体积百分数(70%~10%,间隔5%~10%)下测定*t*_R_,使用双点校正法(DP-RTC)校正^[17]^。根据*k*=(*t*_R_-*t*_0_)/*t*_R_计算保留因子*k*,根据方程(1)建立log *k*-*φ*方程,求得*k*_w_和*S*值,根据方程(2)计算CHI值。采用Origin 9.4进行相关模型建立及数据分析。

## 2 结果与讨论

### 2.1 缓冲盐类型对磺酸化合物保留行为的影响

由于磺酸化合物具有较小的p*K*_a_值(见表1),在实验环境下(pH=7.0)带有完全的负电荷,为典型的强离解化合物,并且磺酸化合物具有明显的紫外特征吸收,可用使用最为普遍的二极管阵列检测器进行检测,因此,我们选择磺酸化合物作为模型化合物。在分析强离解酸性化合物时,四丁基季铵盐为最常使用的离子对试剂,在pH=7.0的条件下,四丁基季铵盐带正电荷,与磺酸化合物形成离子对。实验中采用甲醇作为有机调节剂,固定流动相中离子对试剂为四丁基溴化铵不变,分别以磷酸二氢铵、氯化铵和乙酸铵作为缓冲盐(浓度相同,pH相同),考察缓冲盐类型对磺酸化合物保留行为的影响。

在不同的甲醇比例下,分别获取3种缓冲盐条件下磺酸化合物的*k*值,并建立log *k*-*φ*模型(方程(1)),结果发现,模型的线性相关系数*R*^2^均大于0.99。由log *k*-*φ*模型获取每个化合物在不同流动相下的log *k*_w_和*S*值,并根据方程(2)计算各化合物的CHI值。3种缓冲盐体系下磺酸化合物的log *k*_w_、*S*和CHI值如图1所示。从图1a可以看出,氯化铵体系下溶质的log *k*_w_值最大,而大部分溶质在磷酸二氢铵体系下的log *k*_w_值最小,表明流动相中氯离子的存在有利于增强磺酸化合物的保留。图1b显示大部分溶质在氯化铵体系下的*S*值最大,但SA2、SA4、SA5和SA13在乙酸铵体系下的*S*值明显大于其他两种缓冲盐体系。图1c显示各化合物的色谱疏水指数CHI在乙酸铵体系下最小。尽管在3种缓冲盐体系下的CHI值存在一定差别,但总体上同一化合物在各体系下对应的CHI值近似相等(见图1c)。

**图1:**
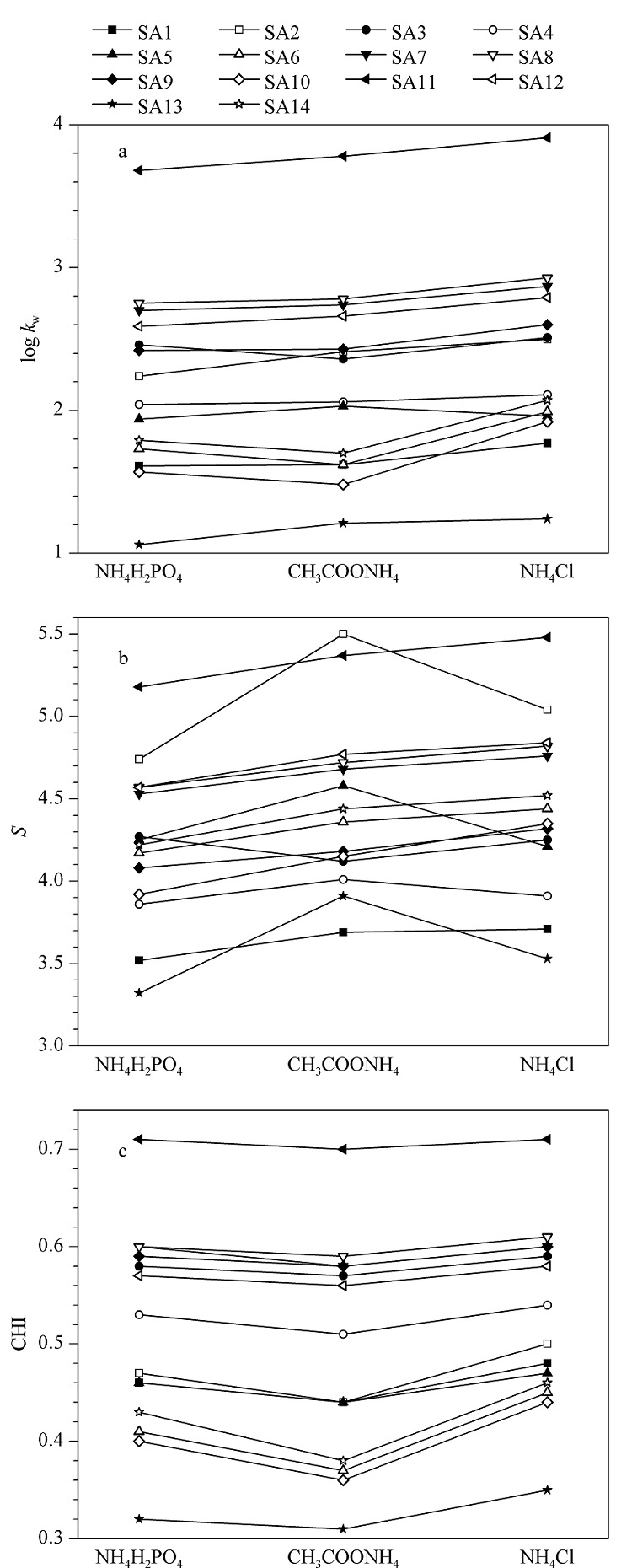
不同缓冲盐体系下磺酸化合物的(a) log *k*_w_、(b) *S*以及(c) CHI

### 2.2 离子对试剂非对离子对磺酸化合物保留行为的影响

以甲醇作为有机调节剂,固定流动相中磷酸二氢铵作为缓冲盐不变,分别采用四丁基溴化铵、四丁基磷酸二氢铵、四丁基硫酸氢铵、四丁基硝酸铵和四丁基乙酸铵作为离子对试剂,考察离子对试剂非对离子对磺酸化合物保留行为的影响。获取5种流动相下化合物的*k*值,建立log *k*-*φ*线性方程,相关系数*R*^2^仍然大于0.99。由log *k*-*φ*线性方程得到各磺酸化合物的log *k*_w_和*S*值,进一步根据方程(2)计算相应的CHI值。使用不同阴离子四丁基季铵盐时,各化合物的log *k*_w_、*S*和CHI值如图2所示。从图2a可以看出,SA8和SA11在Br^-^作为季铵盐阴离子时的log *k*_w_最大,SA3、SA7、SA9和SA12在 ${\mathrm{NO}}_{3}^{-}$ 作为季铵盐阴离子时的log *k*_w_最大,其他8种化合物则在CH_3_COO^-^作为季铵盐阴离子时的log *k*_w_最大。与log *k*_w_不同,几乎所有化合物的*S*值都在CH_3_COO^-^为季铵盐阴离子时最大,并且相对于其他4种阴离子下的*S*值具有明显增强,表明离子对试剂中弱离解性非对离子(乙酸根)的存在有利于增加磺酸化合物的*S*值。*S*是溶质相关的溶剂强度参数,与溶质的疏水表面积相关,反映了溶质和溶剂之间的相互作用。

**图2:**
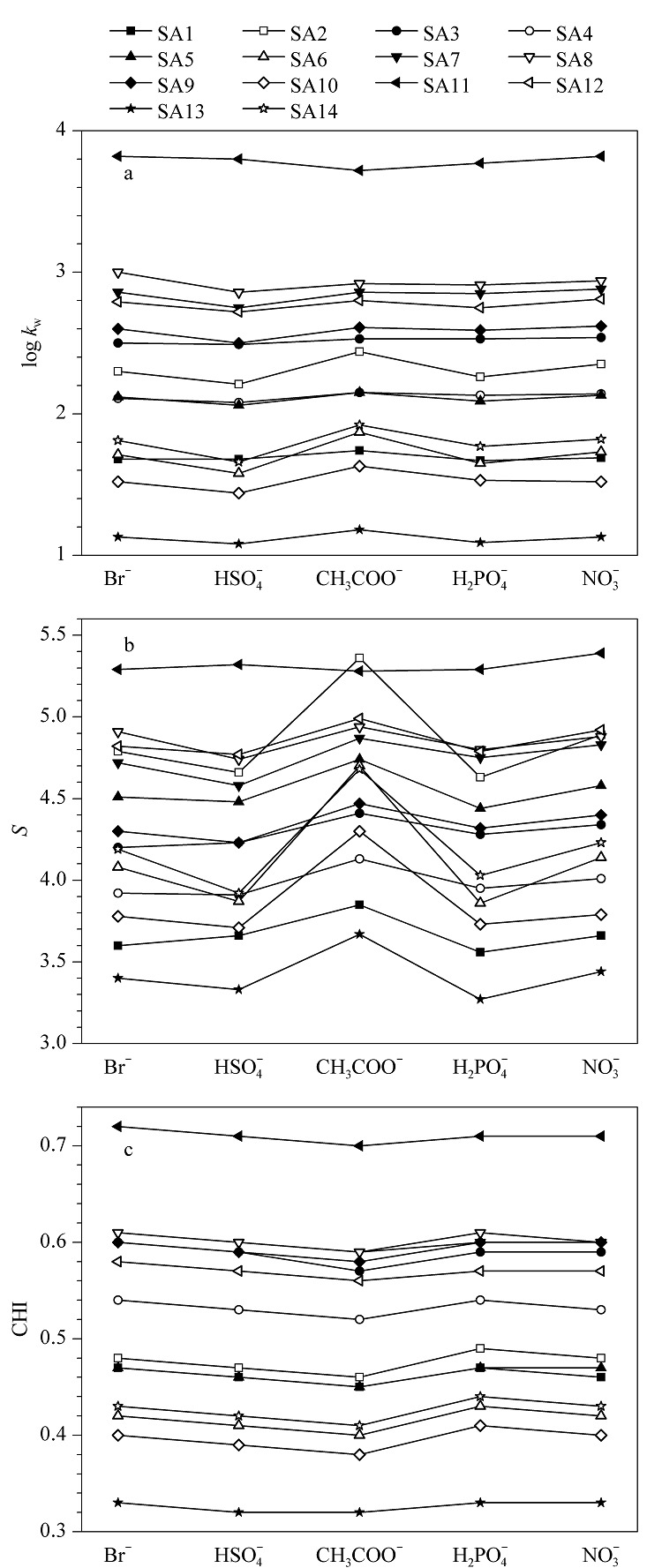
离子对试剂不同阴离子时磺酸化合物的(a) log *k*_w_、 (b) *S*以及(c) CHI

我们的前期研究^[18]^证明,核酸样品的保留在微小的流动相强度变化下产生的巨大变化,与其非常大的*S*值相关。因此,相对于四丁基溴化铵、四丁基磷酸二氢铵、四丁基硫酸氢铵和四丁基硝酸铵,磺酸化合物在四丁基乙酸铵体系下对流动相有机调节剂强度的变化更为敏感。有趣的是,所有化合物的CHI仍然是在CH_3_COO^-^的存在下最小,与缓冲盐类型对磺酸化合物CHI的影响一致。但从CHI数值对比来看,14种磺酸化合物在5种不同的离子对试剂下得到的CHI值相差不大(最大误差值为0.03)。因此,可认为CHI近似相等(见图2c)。以上结果表明,离子对试剂中的阴离子也会影响磺酸化合物的保留行为,对log *k*_w_的影响较为复杂,对*S*值的影响具有一定的共性,对CHI的影响不大。

### 2.3 IP-RPLC的保留机理

对IP-RPLC的保留机理一直存在着两种不同的观点,一种认为是离子对模式保留机理,离子对试剂与带相反电荷的分析物在流动相中首先形成中性物质,然后中性物质分配到疏水性固定相中^[19]^;另外一种认为是动力学离子交换模式保留机理,离子对试剂首先被吸附到固定相上,在固定相表面产生电荷位点,作为带相反电荷分析物的离子交换位点^[20]^。

在动态离子交换模式保留机理中,疏水性的四丁基铵盐可以吸附在固定相表面,形成双电层,磺酸化合物通过与阴离子的交换实现保留,保留主要取决于磺酸化合物所带阴离子电荷的数量。然而,我们的实验表明,具有2个净电荷(*n*_e_=-2)的化合物SA2、SA6、SA10的保留(log *k*_w_)甚至比相同色谱条件下只有1个净电荷(*n*_e_=-1)的化合物(SA7、SA12等)的保留弱(见图1a和2a)。相反,保留最强的化合物为SA11,对应净电荷为-1.14,具有最大的log *D*值(-0.33),即疏水性最强。因此,动态离子交换模式保留机理占主导地位不成立,猜测离子对模式保留机理占主导地位。

在离子对模式保留机理中, 磺酸化合物的保留与和对离子(四丁基铵盐)的结合力以及所形成中性化合物的疏水性有关。以四丁基溴化铵和磷酸二氢铵条件下的保留为例,磺酸化合物log *k*_w_的顺序为SA11>SA8>SA7>SA12>SA3>SA9>SA2>SA4>SA5>SA14>SA6>SA1>SA10>SA13(见图1a),呈现出与磺酸化合物的疏水性(log *D*)、与四丁基铵离子的静电结合能力、空间位阻等多重因素相关的保留。有研究^[21]^表明,在IP-RPLC分析时向流动相中加入与对离子相同电荷的离子会引起溶质保留的变化。Jones等^[21]^在分离不同的硫酸乙酰肝素二糖时, 利用二糖的负电荷与烷基铵离子对试剂的正电荷发生静电相互作用实现保留,通过在流动相中添加2.5 mmol/L铵离子提高了结构相近的硫酸乙酰肝素二糖IIS和IIIS的分离度,并提出丁胺与铵离子之间的竞争是实现这两种异构体分离的主要原因;该研究充分说明,不同的阳离子可以竞争溶质阴离子,从而导致溶质保留的差异。我们的实验中,在不同的阴离子添加剂条件下,非目标阴离子($\mathrm{H}_{2} \mathrm{PO}_{4}^{-}$、 $\mathrm{CH}_{3} \mathrm{COO}^{-}$、 $\mathrm{Cl}^{-}$、 $\mathrm{Br}^{-}$、 $\mathrm{HSO}_{4}^{-}$ 、 $\mathrm{NO}_{3}^{-}$)在流动相中均可以与四丁基铵离子发生静电相互作用,通过与磺酸根竞争形成离子对,竞争效应的强弱应高度依赖于阴离子的结构和化学性质。Dai等^[22]^的研究指出,水对阴离子的水合强度控制着阴离子形成离子对的能力,相对于中等水合的阴离子CH_3_COO^-^(气相离子水合时的吉布斯自由能变Δ*G*=-373 kJ/mol)和Cl^-^(Δ*G*=-347 kJ/mol),高度水合的阴离子$\mathrm{H}_{2} \mathrm{PO}_{4}^{-}$(Δ*G*=-437 kJ/mol)在水中更不易形成离子对,相比之下,“较差”的水合阴离子如$ClO_4^-$(Δ*G*=-214 kJ/mol)在水中更容易形成离子对。基于水合强度,我们推测$\mathrm{H}_{2} \mathrm{PO}_{4}^{-}$与四丁基铵离子的结合能力要明显弱于Cl^-^和CH_3_COO^-^,与磺酸根的竞争力最弱,因此,三者中,在含有$\mathrm{H}_{2} \mathrm{PO}_{4}^{-}$条件下磺酸化合物的保留应该最强。然而,这一推测与我们的实验结果并不相符,图1a显示在Cl^-^存在下各化合物的log *k*_w_最大,图2a则显示大部分化合物在$\mathrm{H}_{2} \mathrm{PO}_{4}^{-}$下的log *k*_w_小于在CH_3_COO^-^下的log *k*_w_值。另外,其他3种阴离子Br^-^、$\mathrm{HSO}_{4}^{-}$和$\mathrm{NO}_{3}^{-}$的实验水合吉布斯自由能变我们无法获取,根据Born离子化模型,电荷相同时,离子半径越大的离子水合吉布斯自由能变越大,对应的竞争力越强。对比图2a中Br^-^、$\mathrm{HSO}_{4}^{-}$和$\mathrm{NO}_{3}^{-}$下的log *k*_w_值,我们发现有的化合物在Br^-^存在下log *k*_w_最大,有的化合物则在$\mathrm{NO}_{3}^{-}$存在下log *k*_w_值最大,并不能得到一致的保留规律。因此,根据非目标阴离子的竞争力强弱以及我们的实验结果表明,磺酸化合物保留过程中并不完全遵循离子对模型保留机理。在两种保留机理下都无法获得与实验结果相一致的结论,因此,我们推测,磺酸化合物保留过程中同时受离子对模型保留机理与动态离子交换模型保留机理控制,以离子对模型保留机理为主。

### 2.4 log *k*_w_、*S*、CHI与log *D*相关性的比较

在IP-RPLC模型下,电荷作用和氢键作用均会影响离解化合物的保留,可以在log *D*-log *k*_w_模型中引入溶质静电荷*n*_e_、氢键酸碱性参数*A*和*B*,建立QSRR模型^[14]^,用于log *D*或保留的预测。在磷酸二氢铵、氯化铵和乙酸铵3种缓冲盐体系下,我们分别以14种磺酸化合物的log *D*_7.0_、log *k*_w_、*n*_e_、*A*和*B*进行多元线性拟合,建立QSRR模型,结果如表2所示。可以看出,3种流动相条件下的log *D*_7.0_和log *k*_w_之间均有良好的线性相关性,*R*^2^达到0.98。为了进一步改善方程的相关性,我们将溶质的极性表面积PSA引入到上述方程中,发现方程的*R*^2^进一步增大,线性得到改善。同样,在引入参数*n*_e_、*A*和*B*的情况下,以*S*对log *D*_7.0_作线性方程,可以得到良好的线性相关性,当引入PSA后,氯化铵体系下的线性相关性得到明显改善,线性相关系数*R*^2^由0.96增大到0.99(见表2)。最后,分别对3种缓冲盐体系下得到的CHI对log *D*_7.0_进行线性拟合,结果显示,PSA引入前后log *D*_7.0_-CHI模型的线性相关性*R*^2^均在0.98以上。表2中的结果表明,溶质的极性表面积PSA会影响溶质的保留,PSA的加入有利于log *D*_7.0_-log *k*_w_、log *D*_7.0_-*S*和log *D*_7.0_-CHI模型的改善。

**表2:** 不同缓冲盐体系下磺酸化合物的log *k*_w_、S、CHI与logD之间的线性关系

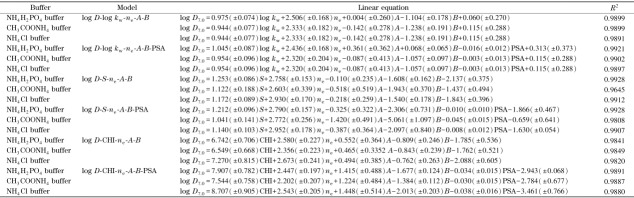

引入溶质静电荷*n*_e_、氢键酸碱性参数*A*和*B*、极性表面积PSA,分别用14种磺酸化合物的log*D*_7.0_值与在5种不同离子对试剂条件下得到的log *k*_w_、*S*以及CHI值,进行多元线性拟合,得到如表3所示的线性方程。可以看出,log *D*_7.0_和log *k*_w_、*S*以及CHI之间均具有良好的线性关系,相关系数*R*^2^均在0.98以上。

**表3:** 不同离子对试剂体系下磺酸化合物的log *k*_w_、S、CHI与log D.o之间的线性关系

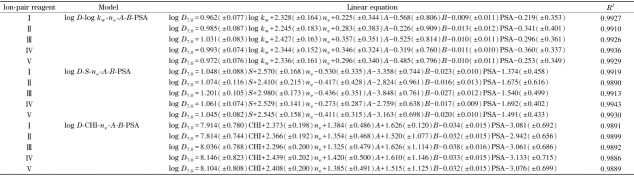

I: tetrabutylammonium bromide; I: tetrabutylammonium hydrogen sulfate; I: tetrabutyl ammonium acetate; IV : tetrabutylammonium hydrogen phosphate; V : tetrabutylammonium nitrate.

用IP-RPLC研究log *D*相关的QSRR模型,通常以log *k*_w_作为疏水性指数,建立log *D*和log *k*_w_之间的线性关系。我们的研究发现,log *D*与*S*以及CHI之间也具有良好的线性相关性。强离解酸性化合物进行IP-RPLC分析时,当流动相中使用不同的缓冲盐阴离子或者离子对试剂阴离子,即使得到的log *k*_w_和*S*值存在一定差异,但最终获取的色谱疏水指数CHI几乎相等。因此,相比log *k*_w_和*S*而言,CHI更适用于QSRR模型的建立。

## 3 结论

本研究发现IP-RPLC中缓冲盐阴离子以及离子对试剂阴离子均会影响磺酸化合物的保留行为。相同离子对试剂情况下,氯化铵体系下的log *k*_w_最大;相同缓冲盐的情况下,离子对试剂中弱离解性阴离子(乙酸根)的存在有利于增加磺酸化合物的*S*值。我们推测磺酸化合物的IP-RPLC保留机理同时存在着离子对模式和动态离子交换模式,并以离子对模式为主。对于强离解酸性化合物,由于在IP-RPLC不同缓冲盐和不同非对离子条件下获得的log *k*_w_和*S*值存在着一定的差异,而CHI值相对稳定,因此CHI更适用于QSRR模型的建立。
